# Utility of circulating cell-free *Mycobacterium tuberculosis* DNA for the improved diagnosis of abdominal tuberculosis

**DOI:** 10.1371/journal.pone.0238119

**Published:** 2020-08-26

**Authors:** Pratibha Sharma, Divya Anthwal, Pooja Kumari, Rakesh Kumar Gupta, Surabhi Lavania, Neera Sharma, Lokesh Kumar Sharma, Deepak Rath, Pavan Kumar Soraganvi, Ashish Sharma, A. K. Gadpayle, R. S. Taneja, Jaya Sivaswami Tyagi, Sagarika Haldar

**Affiliations:** 1 Department of Experimental Medicine and Biotechnology, Post Graduate Institute of Medical Education and Research, Chandigarh, India; 2 Translational Health Science and Technology Institute, NCR Biotech Science Cluster, Faridabad, India; 3 Department of Biotechnology, All India Institute of Medical Sciences, Ansari Nagar, New Delhi, India; 4 Department of Biochemistry, Dr. Ram Manohar Lohia Hospital, New Delhi, India; 5 Department of Medicine, Dr. Ram Manohar Lohia Hospital, New Delhi, India; Institut de Pharmacologie et de Biologie Structurale, FRANCE

## Abstract

Abdominal tuberculosis (ATB) continues to pose a major diagnostic challenge for clinicians due to its nonspecific clinical presentation, variable anatomical location and lack of sensitive diagnostic tools. In spite of the development of several assays till date; no single test has proved to be adequate for ATB diagnosis. In this study, we for the first time report the detection of circulating cell-free *Mycobacterium tuberculosis* (*M*. *tuberculosis*) DNA (cfMTB-DNA) in ascitic fluid (AF) samples and its utility in ATB diagnosis. Sixty-five AF samples were included in the study and processed for liquid culture, cytological, biochemical and molecular assays. A composite reference standard (CRS) was formulated to categorize the patients into ‘Definite ATB’ (*M*. *tuberculosis* culture positive, n = 2), ‘Probable ATB’ (n = 16), ‘Possible ATB’ (n = 13) and ‘Non-TB’ category (n = 34). Two molecular assays were performed, namely, the novel cfMTB-DNA qPCR assay targeting *M*. *tuberculosis devR* gene and Xpert MTB/RIF assay (Xpert), and their diagnostic accuracy was assessed using CRS as reference standard. Clinical features such as fever, loss of weight, abdominal distension and positive Mantoux were found to be strongly associated with ATB disease (p<0.05). cfMTB-DNA qPCR had a sensitivity of 66.7% (95% CI:40.9,86.7) with 97.1% specificity (95% CI:84.7,99.9) in ‘Definite ATB’ and ‘Probable ATB’ group collectively. The sensitivity increased to 70.9% (95% CI:51.9,85.8) in the combined ‘Definite’, ‘Probable’ and ‘Possible’ ATB group with similar specificity. The cfMTB-DNA qPCR assay performed significantly better than the Xpert assay which demonstrated a poor sensitivity of ≤16.7% with 100% (95% CI:89.7,100) specificity (p<0.001). We conclude that cfMTB-DNA qPCR assay is an accurate molecular test that can provide direct evidence of *M*. *tuberculosis* etiology and has promise to pave the way for improving ATB diagnosis.

## Introduction

Tuberculosis (TB) remains a significant global health challenge with an estimate of 10 million people developing the disease globally in 2018 [1]. India alone accounts for 26.9% of this vast global TB burden [1]. Extra-pulmonary TB (EPTB) constitutes ~15–20% of all TB cases in India [2]. Abdominal TB (ATB) is one of the manifestations of EPTB that contributes to 3% of all EPTB cases in India and may involve peritoneal, intestinal, upper gastrointestinal (oesophageal and gastro-duodenal), hepato-biliary, pancreatic and peri-anal sites [3]. ATB remains a major diagnostic challenge owing to the lack of specific clinical features and limited yield of the commonly used diagnostic tests [4]. ATB diagnosis is usually made through a combination of clinical, radiological, microbiological, histological, biochemical and molecular techniques [5]. Conventional microbiological techniques like AFB smear and culture suffer either from low sensitivity (0%-40%) or high turnaround time (culture) which renders them inadequate for appropriate management of the disease [4, 6]. Histopathology demonstrates tuberculous granulomas; however, it alone may not be pathognomonic and requires culture confirmation [7]. Biochemical parameters including ascitic fluid (AF) total protein (>2.5 g/dL), serum-ascites albumin gradient (SAAG) (<1.1 g/dL) and elevated adenosine deaminase (ADA) values are routinely used for the clinical diagnosis of ATB [4]. However, these parameters have a reduced sensitivity in confounding cases of ATB with co-morbidities like liver cirrhosis and chronic liver disease [4, 8–11]. Laparoscopy is the diagnostic tool of choice for ATB and allows inspection of the peritoneum along with providing an option for obtaining biopsies for histology. However, laparoscopic features of ATB may mimic other diseases like sarcoidosis, peritoneal carcinomatosis and Crohn’s disease that necessitates the need of taking biopsies during laparoscopy [4]. Moreover, the procedure (for biopsy) is invasive, expensive and requires competent surgeons and hospitalization [4]. Other tests for ATB diagnosis include immune response-based assays such as the Mantoux test and the IFN-γ release assays; but these tests are usually non-specific and are unable to discriminate between active and latent TB infection [7]. Also, existing commercial serological assays are inconsistent, imprecise and have received a strong negative recommendation by WHO [12].

Various nucleic acid amplification tests (NAATs) including in-house PCR assays have been developed for ATB diagnosis. These assays have targeted different regions of *M*. *tuberculosis* in multiplex (IS*6110*, *devR* and 16S rRNA) or single-plex formats (IS*6110*, 38-kDa protein gene) with a variable sensitivity range of 35%-93% [13–16]. This inconsistent performance of in-house NAATs limits their usefulness for ATB diagnosis. Other NAATs include the Xpert MTB/RIF assay (Xpert) and its advanced version Xpert Ultra, which are WHO-endorsed, automated commercial NAATs, and have proved to be critical tools for the diagnosis of pulmonary TB, extra-pulmonary TB and for children with TB [17, 18]. A small number of studies have reported variable sensitivity (range: 18.3%-100%) for Xpert in diagnosing ATB, with an overall high specificity (range- 90%-100%), limiting its role as a rule-in test [19–22].

Circulating cell-free DNA (cfDNA) is defined as extracellular DNA fragments circulating mainly in various body fluids such as blood, saliva, urine, stool, sputum, various body fluids (cerebrospinal fluid, peritoneal fluid, synovial fluid, lymph and amniotic fluid) and tissues [23]. cfDNA-based tests have been successfully used in detecting cancer, parasitic infections, pathogens like fungi, bacteria, Epstein-Barr virus for nasopharyngeal carcinoma screening and pre-natal diagnosis [23–28]. The presence of cfDNA in peritoneal fluid has been used in diagnosis and prognosis of therapy-related peritoneal degeneration [29]. Detection of circulating cell-free *M*. *tuberculosis* DNA fragments in body fluids has also recently emerged as a novel tool for the diagnosis of TB disease [30–36]. Circulating cell-free *M*. *tuberculosis* DNA (referred as cfMTB-DNA from here onwards) refers to the fragments of cell-free *M*. *tuberculosis* DNA that may have been released from dying human cells and mycobacteria in biological fluids [33]. cfMTB-DNA detection has shown a sensitivity ranging from 50%-79.5% by PCR for pleural TB and 56.5–100% for tuberculous meningitis diagnosis with ≥ 92% specificity in various studies [30–32, 34–37]. To the best of our belief, this is the first study to evaluate the utility of cfMTB-DNA detection in ascitic fluid samples for ATB diagnosis. The objectives of the present study were i) to develop a circulating cell-free *M*. *tuberculosis* DNA probe-based quantitative real-time PCR (qPCR) assay targeting *devR* gene of *M*. *tuberculosis* (cfMTB-DNA qPCR assay) and ii) to compare the performance of the developed assay with the Xpert test for ATB diagnosis.

## Material and methods

### Ethics statement

The ethical approval for this study was obtained from the Institute Ethical Committee of Translational Health Science and Technology Institute (THSTI), Faridabad [THS 1.8.1/(58)], All India Institute of Medical Sciences (AIIMS), New Delhi [IEC/NP-156/2015 RP-13/2014] and Dr. Ram Manohar Lohia Hospital (RMLH), New Delhi [56/2013/IEC/PGIMER-RMLH/9356].

### Study participants

**Fig 1:**
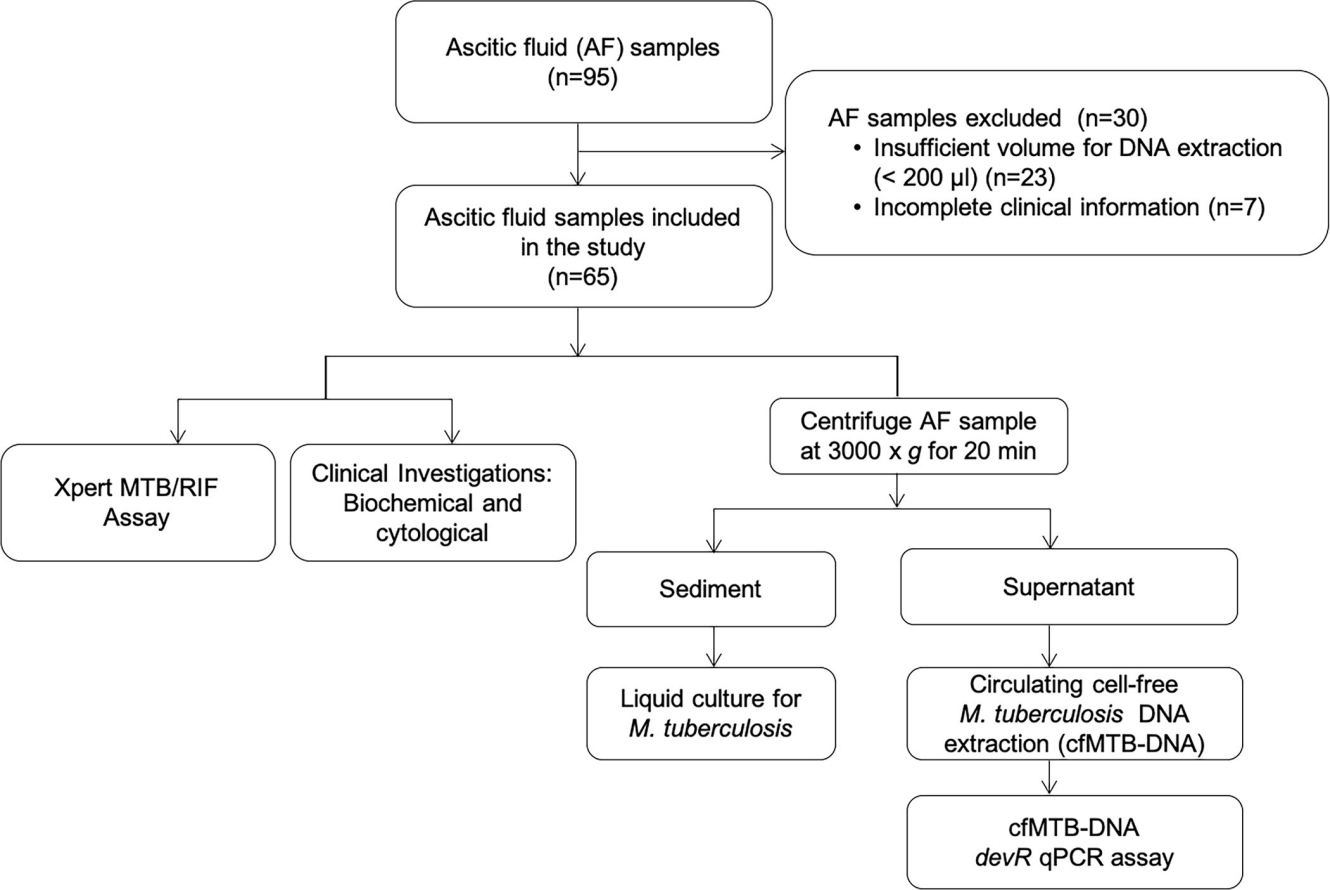
Flowchart of the study.

Patients presenting with ascites (n = 95) visiting the Out-patient Department (OPD) of RMLH were enrolled in this study in a prospective manner. Ascitic fluid samples were collected only after taking written informed consent from patients. No minor cases were included in the study. The samples were collected over a period of 3 years in two phases; March 2014-August 2015 and February 2016-November 2017. Detailed clinical history, radiological findings, biochemical and haematological laboratory investigation results were recorded in a case record form as approved by the Ethics Committee (S1 Appendix). Patients with a past history of tuberculosis (pulmonary TB or extrapulmonary TB) in preceding 2 years or on anti-tubercular therapy (ATT) for > 7 days were excluded. Finally, ascitic fluid samples from 65 patients (age range of 15 to 70 years, 66.2% males and 33.8% females) were included in the study (Fig 1). Patient information was maintained in a classified manner.

**Fig 2:**
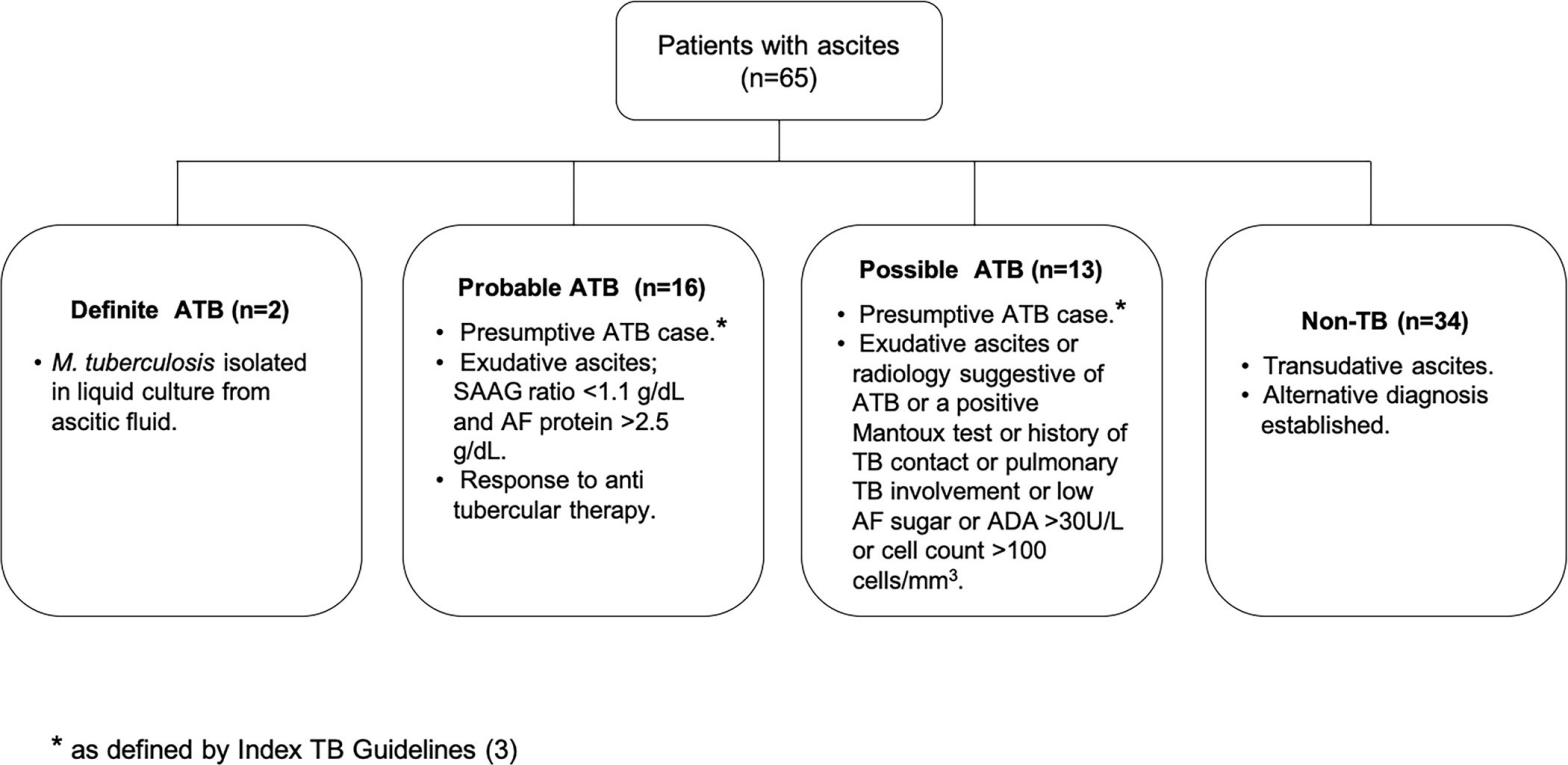
Composite reference standard used for classification of patients enrolled in the study.

The rate of isolation of *M*. *tuberculosis* is very low from ascitic fluid [3], thus a composite reference standard (CRS) was used in the study. The CRS was broadly based on published guidelines and previous categorizations for ATB and comprised of clinical, radiological, microbiological and biochemical findings associated with ATB patients and response to ATT (Fig 2) [3, 22, 38]. Based on the CRS, the patients were categorised into ‘Definite’ ATB, ‘Probable’ ATB, ‘Possible’ ATB and ‘Non-TB’ groups (Fig 2). The ‘Definite’ ATB category included patients who were *M*. *tuberculosis* culture-positive (n = 2) and ‘Probable’ ATB category (n = 16) included patients who were clinically diagnosed as ATB and responded to anti tubercular therapy [3, 38] (Fig 2). ‘Possible’ ATB category (n = 13) included patients who presented with chronic symptoms of ATB with either radiology suggestive of ATB (palpable abdominal mass or omental thickening) or an abnormal chest X-ray (consolidation, calcification or pleural effusion) or presence of other features as described in Fig 2. [3, 4, 22, 38]. However, in this group, the information about response to ATT was not available because either the patient got lost to follow up (n = 11) or left against medical advice (n = 2). Since, the ‘Possible’ ATB category did not fulfil all pre-requisites for ATB classification, we performed a stratified analysis: i) one, including ascitic fluid samples from both ‘Definite’ ATB and ‘Probable’ ATB groups (referred as ‘Definite and Probable’ ATB group) and ii) two, including all three categories collectively i.e., ‘Definite’ ATB, ‘Probable’ ATB and ‘Possible’ ATB’ groups (referred as ‘ATB group’).

### Ascitic fluid collection and processing

Ascitic fluid samples of all patients were subjected to biochemical and cytological investigations at RMLH, New Delhi. The left over ascitic fluid was transferred to the AIIMS laboratory and processed for liquid culture and Xpert on the same day. Briefly, liquid culture for *M*. *tuberculosis* was performed from the sediment obtained after centrifugation of ascitic fluid in Middlebrook 7H9 broth enriched with oleic acid, albumin, dextrose, catalase (OADC) and an antibiotic cocktail; Polymyxin B, Amphotericin B, Nalidixic acid, Trimethoprim and Azlocillin (PANTA) [Becton and Dickinson (BD), Franklin Lakes, New Jersey, United States] (Fig 1). The supernatant was stored at -80°C and used later for cfMTB-DNA extraction (Fig 1). Quantification of cfMTB-DNA was performed targeting the *devR* region of *M*. *tuberculosis* by qPCR.

### DNA extraction and cfMTB-DNA *devR-*based qPCR

Circulating cell-free DNA was isolated using QIAamp® circulating nucleic acid kit (Qiagen, CA, USA) from ascitic fluid supernatant [volume range: 0.2 ml -1.0 ml (mean; 0.67 ml)] as per manufacturer’s instructions and stored at -20 ^o^C (Fig 1). *M*. *tuberculosis* DNA in ascitic fluid was quantified using an in-house TaqMan-based qPCR assay which amplified a specific region of the *devR* gene (139 bp). The designed assay was specific to the *M*. *tuberculosis* complex. Reactions contained 12.5 μl of master-mix (Premix Ex Taq^TM^, Probe qPCR, Takara, Bio, USA, Inc.), 1 μM *devR* 139 forward primer (5’-GCGCTGTCTGATCCTCACGTCC-3’), 0.1 μM *devR* 139 reverse primer (5’-CCGTCCAGCGCCCACATCTTTG-3’) and 0.2 μM *devR* TaqMan^®^ probe (5'-HEX-AGACATCAAGGGAATGGAGTTGGCG-BHQ1-3') and 10 μl sample DNA (cfMTB-DNA). The PCR conditions used were 95°C for 10 min. followed by 40 cycles of 95°C for 30 seconds and 62°C for 30 seconds at which the fluorescence acquisition was determined (CFX 96^TM^ Real-Time PCR Detection system, Bio-Rad, Hercules, California, United States).

### Xpert MTB/RIF assay

The Xpert assay was carried out according to manufacturer's instructions. Briefly, 0.8 ml of ascitic fluid was mixed with sample reagent provided in the assay kit in a ratio of 1:2 and incubated for 15 minutes. 2 ml of the mixture was transferred into the cartridge and inserted in the Xpert instrument for analysis.

### Statistical analysis

The results of all the tests were compiled and analysed after completion of the study in a blinded manner. The performance of cfMTB-DNA qPCR assay and Xpert was evaluated using CRS as the reference standard. DNA amounts in ‘Definite and Probable’ ATB group (true positives) and ‘Non-TB’ group (true negatives) were used to generate receiver operating characteristics (ROC) curves using GraphPad Prism version 5.00 for Windows (GraphPad Software, La Jolla California USA, www.graphpad.com). The cut-off points were derived to achieve test specificity (~97%) consistent with the ‘target product profile’ (TPP) defined for newer diagnostic tests for extrapulmonary tuberculosis [39]. The difference between cfMTB-DNA quantified in the ‘Possible’ ATB group versus other groups (‘Definite’ ATB and ‘Probable’ ATB) was calculated by unpaired t-test (2-tailed) using GraphPad Software. The diagnostic performance parameters for qPCR and Xpert were calculated using web resources (https://www.medcalc.org/calc/diagnostic_test.php). The difference in sensitivity of qPCR and Xpert was determined by McNemar’s test (http://epitools.ausvet.com.au/comparetwotests). The statistical significance of the qualitative and quantitative variables between the ‘ATB’ group and ‘Non-TB’ group was performed by Fisher’s exact test and Mann Whitney tests respectively (the significance was set at a p-value of < 0.05) using GraphPad Software.

## Results

### Clinical and laboratory findings

Of the 95 patients initially enrolled, n = 65 were finally included in the study (Fig 1). Patients were classified into ‘Definite’ ATB (n = 2, *M*. *tuberculosis* culture positive), ‘Probable’ ATB (n = 16), ‘Possible’ ATB (n = 13) and ‘Non-TB’ category (n = 34) on the basis of CRS formulated for the study (Fig 2). The ‘Non-TB’ category included patients of chronic liver disease (n = 26), coronary artery disease with congestive heart failure (n = 3), hepatitis B with ascites (n = 1), chronic kidney disease (n = 1), acute appendicitis (n = 1), acute pancreatitis (n = 1) and malignancy (n = 1). In this study, clinical features such as fever, weight loss, abdominal distension and positive Mantoux was found strongly associated with the ATB disease (p < 0.05) (Table 1). These characteristics were concordant with symptoms generally associated with ATB in other studies [4, 40]. Nineteen percent (6/31) of the ATB category patients had a co-existing pulmonary involvement of TB as evident by an abnormal X-ray finding [cavitation, mediastinal lymphadenopathy, pleural effusion and consolidation]. Six percent (2/31) of the ATB patients had a past history of TB disease and ~29% (9/31) had a history of contact with a TB patient. In the ATB category, 26% (8/31) of the patients showed hepatomegaly, followed by splenomegaly (16%; 5/31), necrotic and mesenteric lymph nodes (23%; 7/31), intramural and omental thickening (10%; 3/31) and tubovarian mass (3%; 1/31) on the ultrasound scans apart from moderate to massive ascites. *M*. *tuberculosis* culture was positive in 6.5% (2/31) of ATB patients. Various laboratory investigations performed in all patients of the study are described in Table 2. Ascitic fluid total protein and ADA values were significantly higher in the ‘ATB group’ vs. Non-TB group (p < 0.05); whereas, SAAG ratios were significantly lower (p < 0.05) (Table 2)

**Table 1:** Characteristics and clinical features of the study participants^#^.

Clinical features	‘Definite’ ATB (n = 2)	‘Probable’ ATB (n = 16)	‘Possible’ ATB (n = 13)	‘ATB’ group^$^ (n = 31)	‘Non-TB’ group (n = 34)	p-value*
**Male/Female (M%/F%)**	2/0 (100/0)	6/10 (37.5/62.5)	13/0 (100/0)	21/10 (67.7/32.2)	22/12 (64.7/35.2)	-
**Fever**	2 (100)	14 (87.5)	8 (61.5)	24 (77.4)	10 (29.4)	**0.001**
**Headache**	0 (0)	1 (6.2)	1 (7.6)	2 (6.4)	1 (2.94)	0.601
**Vomiting**	2 (100)	4 (25)	5 (38.4)	11 (35.4)	8 (23.5)	0.413
**Loss of appetite**	2 (100)	11 (68.7)	9 (69.2)	21 (67.7)	18 (52.9)	0.311
**Constipation**	2 (100)	7 (43.7)	4 (30.7)	13 (41.9)	10 (29.4)	0.312
**Diarrhoea**	0 (0)	2 (12.5)	1 (7.6)	3 (9.6)	2 (5.8)	0.663
**Weight loss**	2 (100)	10 (62.5)	5 (38.4)	17 (54.8)	7 (20.5)	**0.005**
**Night Sweats**	1 (50)	2 (12.5)	3 (23.0)	6 (19.3)	3 (8.8)	0.290
**Abdominal distension**	0 (0)	5 (31.2)	3 (23.0)	8 (25.8)	2 (5.9)	**0.038**
**Abdominal pain**	1 (50)	11 (68.7)	8 (61.5)	20 (64.5)	25 (73.5)	0.591
**Cough**	1 (50)	6 (37.5)	4 (30.7)	11 (35.4)	9 (26.4)	0.591
**Chest pain**	0 (0)	3 (18.7)	1 (7.6)	4 (12.9)	4 (11.7)	1.000
**Haemoptysis**	0 (0)	2 (12.5)	2 (15.3)	4 (12.9)	1 (2.9)	0.183
**Past history of TB**	0 (0)	1 (6.2)	1 (7.6)	2 (6.4)	1 (2.9)	0.601
**History of TB contact**	1 (50)	4 (25)	4 (30.7)	9 (29.0)	7 (20.5)	0.566
**Alcoholism**	1 (50)	1 (6.2)	5 (38.4)	7 (22.5)	15 (44.1)	0.114
**Smoking**	0 (0)	1 (6.2)	5 (38.4)	6 (19.3)	5 (14.7)	0.744
**Amenorrhea**	0 (0)	5 (31.2)	0	5 (16.1)	4 (11.7)	0.726
**Pallor**	2 (100)	5 (31.2)	5 (38.4)	12 (38.7)	13 (38.2)	1.000
**Pedal edema**	0 (0)	3 (18.7)	2 (15.3)	5 (16.1)	8 (23.5)	0.543
**Hepatomegaly**	1 (50)	4 (25)	3 (23.0)	8 (25.8)	7 (20.5)	0.769
**Splenomegaly**	1 (50)	1 (6.2)	3 (23.0)	5 (16.1)	3 (8.8)	0.463
**Positive Mantoux test^**	1/2 (50)	6/14 (42.8)	2/8 (25.0)	9/24 (37.5)	1/17 (5.8)	**0.028**

^#^values denoted as number of patients showing clinical signs in each category; values in brackets denotes the percentage of patients presenting the clinical features.

^$^ATB group includes ‘Definite’, ‘Probable’ and ‘Possible ATB’ categories collectively.

^Mantoux test data was available for n = 24 patients in ATB group and n = 17 patients in non-TB group.

*statistical significance calculated between ‘ATB’ group vs. Non-TB group by Fisher’s test, significant values (*p*-value < 0.05) are in bold.

**Table 2:** Laboratory findings in enrolled study participant’s^#^.

Parameters	‘Definite’ ATB (n = 2)	‘Probable’ ATB (n = 16)	‘Possible’ ATB (n = 13)	‘ATB’ group$ (n = 31)	‘Non-TB group’ (n = 34)	p-value*
**Haemoglobin (g/dL)**	7.8 (6.1–9.6)	9 (8–11.3)	9.2 (5.2–10.9)	9.1 (6.7–10.9)	8.2 (7.3–10.3)	0.994
**TLC**	9900 (9800–10000)	7200 (5600–9700)	9900 (6800–13000)	8100 (6150–11725)	11000 (8000–14600)	0.078
**ESR (mm/hr)**	22 (10–34)	27 (15.5–42.5)	17 (12.7–24.7)	22 (14–33.5)	21 (12–34)	0.604
**Direct bilirubin (mg/dL)**	0.3 (0.2–0.4)	0.5 (0.3–0.7)	0.5 (0.3–2.2)	0.4 (0.3–0.8)	1.3 (0.2–2.2)	0.158
**Indirect bilirubin (mg/dL)**	0.55 (0.4–0.7)	0.5 (0.4–0.7)	0.6 (0.6–2.0)	0.6 (0.4–0.9)	1.3 (0.4–2.4)	0.086
**AST (U/L)**	298 (44–551)	47.5 (37–51.5)	48 (32.7–151)	47.5 (34.2–124.2)	62 (45–109)	0.581
**ALT (U/L)**	88.5 (31–146)	36 (25–43)	47 (36–125.5)	42 (28–89)	46.5 (28–73)	0.978
**ALP (U/L)**	193 (184–201)	165 (111–193)	134 (119–312)	140 (113–201)	135 (96–200)	0.366
**Urea (mg/dL)**	49.5 (43–56)	36.5 (27.2–41)	51 (36–84)	41 (34–56)	66 (41–117)	**0.013**
**Creatinine (mg/dL)**	1 (0.9–1.1)	0.8 (0.7–1.0)	1.2 (0.9–1.6)	0.9 (0.8–1.2)	1.3 (0.9–2.4)	**0.026**
**Serum albumin (g/dL)**	2.7 (2.4–3.1)	3 (2.3–3.4)	2.5 (2–2.6)	2.6 (2.3–3.0)	2.4 (2–2.7)	0.083
**Serum globulin (g/dL)**	4.2 (4.1–4.3)	3.1 (2.4–3.7)	3.5 (2.8–3.7)	3.4 (2.5–3.9)	3.6 (3.3–4.3)	0.067
**AF Cytology (cu. mm)**	175 (150–200)	250 (90–350)	200 (150–600)	200 (150–350)	100 (50–275)	0.154
**AF Sugar (mg/dL)**	141 (78–205)	90 (76–97)	99 (61–109.5)	93 (73.2–104)	108 (84–142)	**0.030**
**AF Protein (g/dL)**	1 (0.8–1.2)	3.9 (2.9–5.0)	2.2 (1.9–2.9)	2.9 (2.1–4.2)	1.8 (1.1–2.7)	**0.027**
**Blood sugar (mg/dL)**	NA	78 (71.5–113)	112 (86–137)	90 (73–126)	102 (83–140)	0.260
**SAAG’s Ratio (g/dL)**	2.0 (1.9–2.2)	0.9 (0.5–1)	1.3 (1–1.6)	1.0 (0.7–1.6)	1.7 (1.3–1.9)	**0.003**
**ADA (U/L)**	38.9 (12–65.8)	41 (28–46.7)	28.0 (11.1–32.8)	33.3 (12.2–41.6)	7.7 (4.6–10.4)	**0.001**
**Uric acid (mg/dL)**	7.2 (3.3–11.1)	6.0 (4–9)	4.7 (3.2–6.6)	5.9 (3.3–8.8)	7.0 (6–9.5)	**0.047**

^#^Values indicated as median of the laboratory findings in each category; numbers in brackets denotes the 1^st^ and 3^rd^ quartile values [except in Definite ATB group (n = 2), where it denotes range].

^$^ATB group includes ‘Definite’, ‘Probable’ and ‘Possible’ ATB categories collectively.

*statistical significance calculated between ‘ATB’ group vs. ‘Non-TB group using Mann Whitney’s test, significant values (*p*-value < 0.05) are in bold. NA- data not available. TLC- total leucocyte count, ESR- erythrocyte sedimentation rate, AST- Aspartate amino transferase, ALT- Alanine amino transferase, ALP- Alkaline phosphatase, AF- ascitic fluid, SAAG’s ratio- serum ascites albumin gradient ratio, ADA- adenosine deaminase assay.

### Performance outcome of cMTB-DNA *devR*-qPCR assay

**Fig 3:**
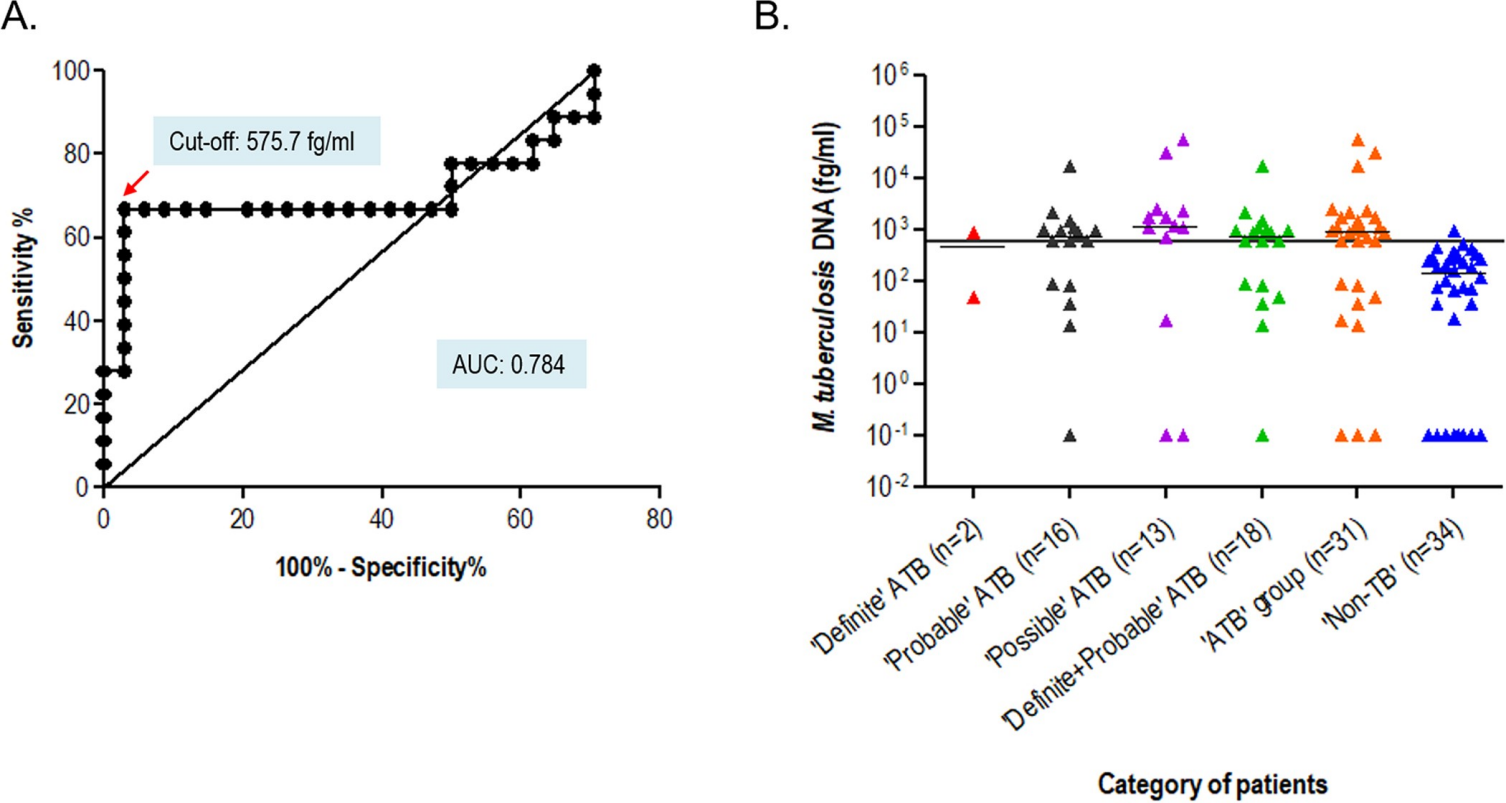
Detection of circulating cell-free *M*. *tuberculosis* DNA in ascitic fluid samples by cfMTB-DNA qPCR assay. A.) ROC curve generated from the cell-free MTB-DNA amount quantified from ascitic fluid samples of ‘Definite and Probable’ ATB vs. ‘Non-TB’ patients. B.) Scatter plot of *M*. *tuberculosis* (fg/ml) in each categorized group. The black horizontal line across the scatter plot denotes the cut-off value of 575.7 fg/ml set by ROC curve analysis of ‘Definite and Probable ATB’ group (combined) *vs*. ‘Non-TB’ disease group. The black line in each data set denotes the median value.

Circulating cell-free *M*. *tuberculosis* DNA extracted from ascitic fluid samples were quantified using the *devR*-qPCR assay. Total DNA amounts in ascitic fluid from ‘Definite and Probable ATB’ group (true positives) and ‘Non-TB’ group (true negatives) were used to construct ROC curves and cut-off value was set at 575.7 fg/ml (equivalent to 115 *M*. *tuberculosis* bacterial genome equivalents) to attain a specificity of 97% (Fig 3A). cfMTB-DNA *devR*-qPCR assay yielded a sensitivity of 66.7% (95% CI: 40.9, 86.7) in ‘Definite and Probable ATB’ category with 97.1% (95% CI: 84.7, 99.9) specificity (Table 3). The sensitivity increased to 70.9% (95% CI: 51.9, 85.8; Table 3, Fig 3) in ‘ATB’ group (‘Definite’, ‘Probable’ and ‘Possible ATB’ groups collectively) with no decrease in the specificity (97.1%). Notably, one out of two ‘Definite ATB’ samples (n = 2) was positive by the cfMTB-DNA *devR*-qPCR assay.

**Table 3:** Diagnostic accuracy of cfMTB-DNA qPCR assay and Xpert MTB/RIF assay.

Assays	TP	FP	TN	FN	Sensitivity* %	Specificity* %	PPV*^ %	NPV*^ %	(+) LR*^	(-) LR*^
*devR*-qPCR assay (‘Definite & Probable’ ATB)	12	1	33	6	66.7 (40.9;86.7)	97.1 (84.7;99.9)	92.3 (62.9;98.4)	84.6 (74.1;91.3)	22.7 (3.2;160.7)	0.34 (0.2;0.7)
*devR*-qPCR assay (‘ATB’ group)	22	1	33	9	70.9 (51.9;85.8)	97.1 (84.7;99.9)	97.7 (75.9;99.3)	78.6 (67.8;86.4)	24.1 (3.5;168.6)	0.30 (0.17;0.52)
Xpert (‘Definite & Probable’ ATB)	3	0	34	15	16.7 (3.6;41.4)	100 (89.7;100)	100^#^	69.4 (64.8;73.6)	-	0.83 (0.7–1.0)
Xpert (‘ATB’ group)	3	0	34	28	9.7 (2.04;25.8)	100 (89.7;100)	100^#^	54.8 (52;57.7)	-	0.90 (0.8;1.0)
Xpert + *devR* qPCR assay (‘Definite & Probable’ ATB group)	13	1	33	5	72.2 (46.5;90.3)	97.1 (84.7;99.9)	92.8 (64.9;98.9)	86.8 (75.8;93.3)	24.6 (3.5;173)	0.29 (0.14;0.60)
Xpert + *devR* qPCR assay (‘ATB’ group)	23	1	33	8	74.2 (55.4;88.1)	97.1 (84.7;99.9)	95.8 (76.7;99.4)	80.5 (69.4;88.3)	25.2 (3.6;176)	0.27 (0.15;0.48)

*value in brackets indicates 95% confidence interval.

^#^95% confidence interval not calculable.

^PPV- positive predictive value, NPV- negative predictive value, (+) LR- positive likelihood ratio and (-) LR- negative likelihood ratio.

### Performance outcome of Xpert MTB/RIF assay

Xpert was performed on all ascitic fluid samples. The sensitivity of Xpert was 16.7% (95% CI: 3.6, 41.4) in the ‘Definite and Probable’ ATB group with 100% (95% CI: 89.7,100) specificity. Xpert detected 3/18 ‘ATB’ patients (1 from ‘Definite’ ATB category and 2 from ‘Probable’ ATB category). However, in the combined ‘ATB’ group (‘Definite’ ATB, ‘Probable’ ATB and ‘Possible’ ATB), the sensitivity of Xpert decreased to 9.7% (95% CI: 2.04, 25.8) (as no sample was positive in ‘Possible’ ATB category) with no decrease in specificity (Table 3). No rifampicin resistance was detected in any sample.

### Comparative evaluation of Xpert *vs*. cfMTB-DNA qPCR assay

The sensitivity of detection of circulating cell-free *M*. *tuberculosis* DNA by cfMTB-DNA qPCR assay was 66.7% as compared to 16.7% by Xpert in the ‘Definite and Probable’ ATB combined group (Table 3). The difference was further enhanced when the ‘Possible’ ATB group was included in the analysis (70.9% *vs*. 9.7%). The sensitivity increment of cfMTB-DNA qPCR over Xpert was statistically significant (p<0.001). Combining the results of Xpert and cfMTB-DNA qPCR yielded a sensitivity of 72.2% (95% CI: 46.5,90.3) in ‘Definite and Probable’ ATB category with 97.1% (95% CI: 84.7,99.9) specificity. The sensitivity was further enhanced to 74.2% (95% CI: 55.4,88.1) in the combined ‘ATB’ group with similar specificity (Table 3).

## Discussion

ATB is a clinical challenge because of its non-specific clinical presentation and lack of accurate diagnostic tools [4, 41]. In spite of the availability of several diagnostic platforms; no test has proved to be solely adequate for diagnosing ATB [4, 19, 42]. The detection of circulating cell-free *M*. *tuberculosis* DNA (cfMTB-DNA) has recently emerged as a tool for diagnosing paucibacillary forms of TB such as tuberculous meningitis and pleural TB [30–32, 34–37]. In the present study, to the best of our knowledge, we for the first time assessed the utility of cfMTB-DNA for ATB diagnosis.

The developed cfMTB-DNA *devR*-based qPCR assay demonstrated a sensitivity of 66.7% (95% CI:40.9,86.7) collectively in the ‘Definite and Probable’ ATB group and 70.9% (95% CI: 51.9,85.8) in the combined ‘ATB’ group (‘Definite’, ‘Probable’ and ‘Possible’ ATB). Nine (9/31) samples were false negative by cfMTB-DNA qPCR assay in the combined ‘ATB group’ which included one (1/2) from ‘Definite’ ATB group, five (5/16) from ‘Probable’ ATB group and three (3/13) from the ‘Possible’ ATB group. The false negative qPCR results might be attributed to the varying amounts of cfMTB-DNA available in ascitic fluid for amplification. Although, a few qPCR results were positive with very low volumes of ascitic fluid (~41% positive ascitic fluid samples had a volume of <300 μl), we believe that the false negative qPCR results might be reduced by using a larger volume of ascitic fluid for cfMTB-DNA extraction. The volume range of ascitic fluid used in this study was in the range of 200 μl to 1 ml (depending on sample volume availability), and we believe that an increase in sample volume can ensure optimum amount of cfDNA extraction which will lead to fewer false negative results [43]. An improved sensitivity was obtained for urinary EGFR cfDNA detection in NSCLC patients when ~90–100 ml of urine was used for analysis [44]. The specificity for the cfMTB-DNA qPCR assay was 97.1% (95% CI: 84.7,99.9) in both ‘Definite and Probable’ ATB group and combined ‘ATB’ group. One sample (1/34) was false positive in this study. The diagnosis of this patient was chronic liver disease (CLD) and it might be possible that the false positive qPCR result was due to an underlying ATB disease that was masked by CLD; as it has been reported that in countries with high TB prevalence there is a high possibility of developing ATB disease in CLD patients [45, 46].

The origin of cfDNA in the circulation is believed to be either through apoptosis, necrosis, phagocytosis, active secretion, exocytosis or breakdown of pathogens and the increase in the amount of cfDNA released has been correlated with the increase in the dead and dying cells [47–51]. This released cfDNA is further cleared via blood, body fluids, liver, spleen, kidney or lymph nodes [52–54] by immune system mainly by macrophages [55, 56]. In healthy individuals, the cfDNA and apoptotic cells are rapidly cleared; thus leading to a reduced level of cfDNA in circulation [47, 52]; however, during chronic inflammation, excessive cell death and pathological conditions, there is an insufficient clearance of cfDNA leading to its accumulation in circulation [47, 52]. This insufficient clearance might explain the accumulation of cfDNA in pathological conditions (here abdominal TB) [47, 52]. The actual levels of cfDNA in circulation relies upon the balance between the DNA release and DNA clearance processes and this inter-play between them marks the actual levels of cfDNA in the circulation [52]. It was observed in the present study that the mean DNA amount quantified by the cfMTB-DNA qPCR assay in ascitic fluid was higher in the ‘Possible’ ATB group (Mean 7492 fg/ml ± 4545) followed by ‘Probable’ ATB group (Mean 1754 fg/ml ± 1068) and ‘Definite’ ATB group (Mean 468.1 fg/ml ± 417.9). The difference between circulating cfMTB-DNA amount quantified in the ‘Possible’ ATB group versus combined group of ‘Definite and Probable’ ATB was highly significant (p < 0.0001). This observation proves a reverse relationship between the presence of number of intact *M*. *tuberculosis* bacilli and circulating cfMTB-DNA, i.e. greater is the number of intact bacilli (which is expected in ‘Definite’ group), lower is the amount of circulating cfMTB-DNA. This significant difference between the mean DNA amounts between different categories might be because of different cellular fate (death modality) of *M*. *tuberculosis* infected macrophages which influences the infection outcome [57]. During apoptosis or necrosis of *M*. *tuberculosis* infected macrophages, the viability of *M*. *tuberculosis* bacilli is reduced whereas cfMTB-DNA release is increased in the circulation [49, 57] which might be the reason for increased cfMTB-DNA in the ‘Probable and Possible ATB’ categories. However, in the ‘Definite ATB’ group the reduced amount of cfMTB-DNA might be due to the inhibition of apoptosis of *M*. *tuberculosis* infected macrophages as one of the virulence mechanisms against host defence which allows *M*. *tuberculosis* to replicate [57], thereby leading to more number of intact bacilli and not as much amount of cfMTB-DNA.

Xpert exhibited a modest sensitivity of 16.7% and 9.7% in the combined group of ‘Definite and Probable’ ATB and combined ‘ATB’ (‘Definite’, ‘Probable’ and ‘Possible’) group, respectively in the present study. One study reported a similar sensitivity of ~18.3% for Xpert against CRS [21]; however, a higher sensitivity of 32% and 50% has also been reported [22, 58]. In microbiologically confirmed ATB cases, a comparatively higher sensitivity has been reported for Xpert. A pooled sensitivity of 59% (Range:33%-100%) has been recently described by WHO which was similar to the sensitivity of Xpert in definite ATB cases in our study (50%), albeit we had only 2 cases [20]. We believe that since Xpert is based on the principle of detecting ‘genomic DNA’ from intact *M*. *tuberculosis* bacilli (which is expected to be higher in microbiologically confirmed ATB cases), the sensitivity of Xpert is higher in ‘Definite’ ATB samples. In CRS confirmed cases, intact *M*. *tuberculosis* bacilli are expected to be fewer, leading to a higher sensitivity of cfMTB-DNA qPCR assay vs. Xpert. However, the excellent specificity (100%) of Xpert provided credence to a sample being not labelled as false positive and indicated its utility as a rule in test [59].

The major strength of our study was the development of a cfMTB-DNA based assay and establishing its utility in ATB diagnosis. cfDNA is an attractive biomarker for TB diagnosis that indicates the presence of pathogen in the system [33]. This approach enabled utilization of both the sediment and supernatant fractions of ascitic fluid for *M*. *tuberculosis* culture (reference standard) and circulating cell-free DNA based tests, respectively. The limitations of this study were small sample size, limited sample volumes and identification of only two ‘Definite’ ATB patients. Overall, cfMTB-DNA qPCR assay showed a better diagnostic accuracy for ATB as compared to culture and Xpert, which indicates its diagnostic utility in ATB diagnosis. However, the assay missed one culture positive and one Xpert-positive patient which might be because of the inverse relationship between the disease category and the amount of genomic DNA. Therefore, we believe that tests based on both genomic DNA and circulating DNA should be performed on ATB samples, as the combinatorial outcome of both these formats will enable us to diagnose both Definite and Probable/Possible forms of ATB. By combining our novel cfMTB-DNA qPCR assay with Xpert, the overall diagnostic sensitivity in ‘Definite and Probable’ ATB group increased to 72.2%, with no decrease in the specificity. When test results of ‘Possible’ ATB category were also included, the combined sensitivity of qPCR and Xpert increased to 74.2%. The ‘Possible’ ATB category was formulated specifically for this study, we believe that the inclusion of this category and the detection of subjects belonging to it highlights the diagnostic challenges that plague ATB. These patients possibly had TB which was missed due to lack of optimal diagnostic tools [46].

The detection of cfMTB-DNA in the ascitic fluid of ATB patients has proved to be a promising diagnostic tool. We conclude that the developed cfMTB-DNA qPCR assay has an immense potential for the diagnosis of ATB and can also be adapted to other paucibacillary forms of EPTB. Nevertheless, for the application of this assay in the clinic, we will require its validation on a larger number of samples.

## Supporting information

S1 Appendix (PDF)

## References

[1] WHO. Global Tuberculosis Report. 2019.

[2] Sharma SK, Mohan A. Extrapulmonary tuberculosis. The Indian journal of medical research. 2004;120(4):316–53. Epub 2004/11/03. 15520485

[3] WHO. Index TB- Guidelines for Extra Pulmonary tuberculosis India. 2016.

[4] Sanai FM, Bzeizi KI. Systematic review: tuberculous peritonitis—presenting features, diagnostic strategies and treatment. Alimentary pharmacology & therapeutics. 2005;22(8):685–700. Epub 2005/10/04. 16197489 10.1111/j.1365-2036.2005.02645.x

[5] Sharma MP, Bhatia V. Abdominal tuberculosis. The Indian journal of medical research. 2004;120(4):305–15. Epub 2004/11/03. 15520484

[6] Singh V, Kumar P, Kamal J, Prakash V, Vaiphei K, Singh K. Clinicocolonoscopic profile of colonic tuberculosis. The American journal of gastroenterology. 1996;91(3):565–8. Epub 1996/03/01. 8633510

[7] Rathi P, Gambhire P. Abdominal Tuberculosis. The Journal of the Association of Physicians of India. 2016;64(2):38–47. Epub 2016/10/13. 27730779

[8] Shakil AO, Korula J, Kanel GC, Murray NG, Reynolds TB. Diagnostic features of tuberculous peritonitis in the absence and presence of chronic liver disease: a case control study. The American journal of medicine. 1996;100(2):179–85. Epub 1996/02/01. 10.1016/s0002-9343(97)89456-9 8629652

[9] Demir K, Okten A, Kaymakoglu S, Dincer D, Besisik F, Cevikbas U, et al Tuberculous peritonitis—reports of 26 cases, detailing diagnostic and therapeutic problems. European journal of gastroenterology & hepatology. 2001;13(5):581–5. Epub 2001/06/09. 11396540 10.1097/00042737-200105000-00019

[10] Vardareli E, Kebapci M, Saricam T, Pasaoglu O, Acikalin M. Tuberculous peritonitis of the wet ascitic type: clinical features and diagnostic value of image-guided peritoneal biopsy. Digestive and liver disease: official journal of the Italian Society of Gastroenterology and the Italian Association for the Study of the Liver. 2004;36(3):199–204. Epub 2004/03/30. 10.1016/j.dld.2003.10.016 15046190

[11] Hillebrand DJ, Runyon BA, Yasmineh WG, Rynders GP. Ascitic fluid adenosine deaminase insensitivity in detecting tuberculous peritonitis in the United States. Hepatology. 1996;24(6):1408–12. Epub 1996/12/01. 10.1002/hep.510240617 8938171

[12] WHO. Commercial Serodiagnostic Tests for Diagnosis of Tuberculosis. Policy Statement. 2011. 26158189

[13] Hallur V, Sharma M, Sethi S, Sharma K, Mewara A, Dhatwalia S, et al Development and evaluation of multiplex PCR in rapid diagnosis of abdominal tuberculosis. Diagnostic microbiology and infectious disease. 2013;76(1):51–5. Epub 2013/04/24. 10.1016/j.diagmicrobio.2013.02.022 23608350

[14] Yonal O, Hamzaoglu HO. What is the most accurate method for the diagnosis of intestinal tuberculosis? The Turkish journal of gastroenterology: the official journal of Turkish Society of Gastroenterology. 2010;21(1):91–6. Epub 2010/06/17. 20549889 10.4318/tjg.2010.0063

[15] Kulkarni S, Vyas S, Supe A, Kadival G. Use of polymerase chain reaction in the diagnosis of abdominal tuberculosis. Journal of gastroenterology and hepatology. 2006;21(5):819–23. Epub 2006/05/18. 10.1111/j.1440-1746.2006.04030.x 16704529

[16] Portillo-Gomez L, Morris SL, Panduro A. Rapid and efficient detection of extra-pulmonary Mycobacterium tuberculosis by PCR analysis. The international journal of tuberculosis and lung disease: the official journal of the International Union against Tuberculosis and Lung Disease. 2000;4(4):361–70. Epub 2000/04/25. 10777087

[17] Steingart KR, Schiller I, Horne DJ, Pai M, Boehme CC, Dendukuri N. Xpert(R) MTB/RIF assay for pulmonary tuberculosis and rifampicin resistance in adults. The Cochrane database of systematic reviews. 2014(1):CD009593 Epub 2014/01/23. 10.1002/14651858.CD009593.pub3 24448973 PMC4470349

[18] WHO. Rapid Communication: Molecular assays as initial tests for the diagnosis of tuberculosis and rifampicin resistance. 2020.

[19] Kohli M, Schiller I, Dendukuri N, Dheda K, Denkinger CM, Schumacher SG, et al Xpert((R)) MTB/RIF assay for extrapulmonary tuberculosis and rifampicin resistance. The Cochrane database of systematic reviews. 2018;8:CD012768 Epub 2018/08/28. 10.1002/14651858.CD012768.pub2 30148542 PMC6513199

[20] WHO. WHO consolidated guidelines on tuberculosis. Module:3, Rapid diagnosis of tuberculosis detection. 2020. 33999549

[21] Liu R, Li J, Tan Y, Shang Y, Li Y, Su B, et al Multicenter evaluation of the acid-fast bacillus smear, mycobacterial culture, Xpert MTB/RIF assay, and adenosine deaminase for the diagnosis of tuberculous peritonitis in China. International journal of infectious diseases: IJID: official publication of the International Society for Infectious Diseases. 2020;90:119–24. Epub 2019/11/07. 31693941 10.1016/j.ijid.2019.10.036

[22] Tadesse M, Abebe G, Bekele A, Bezabih M, Yilma D, Apers L, et al Xpert MTB/RIF assay for the diagnosis of extrapulmonary tuberculosis: a diagnostic evaluation study. Clinical microbiology and infection: the official publication of the European Society of Clinical Microbiology and Infectious Diseases. 2019;25(8):1000–5. Epub 2018/12/26. 10.1016/j.cmi.2018.12.018 30583052

[23] Weerakoon KG, McManus DP. Cell-Free DNA as a Diagnostic Tool for Human Parasitic Infections. Trends in parasitology. 2016;32(5):378–91. Epub 2016/02/06. 10.1016/j.pt.2016.01.006 26847654

[24] De Vlaminck I, Martin L, Kertesz M, Patel K, Kowarsky M, Strehl C, et al Noninvasive monitoring of infection and rejection after lung transplantation. Proceedings of the National Academy of Sciences of the United States of America. 2015;112(43):13336–41. Epub 2015/10/16. 10.1073/pnas.1517494112 26460048 PMC4629384

[25] Schwarzenbach H, Hoon DS, Pantel K. Cell-free nucleic acids as biomarkers in cancer patients. Nature reviews Cancer. 2011;11(6):426–37. Epub 2011/05/13. 10.1038/nrc3066 21562580

[26] Lo YM, Corbetta N, Chamberlain PF, Rai V, Sargent IL, Redman CW, et al Presence of fetal DNA in maternal plasma and serum. Lancet. 1997;350(9076):485–7. Epub 1997/08/16. 10.1016/S0140-6736(97)02174-0 9274585

[27] Dinakaran V, Rathinavel A, Pushpanathan M, Sivakumar R, Gunasekaran P, Rajendhran J. Elevated levels of circulating DNA in cardiovascular disease patients: metagenomic profiling of microbiome in the circulation. PloS one. 2014;9(8):e105221 Epub 2014/08/19. 10.1371/journal.pone.0105221 25133738 PMC4136842

[28] Lo YM, Chan LY, Lo KW, Leung SF, Zhang J, Chan AT, et al Quantitative analysis of cell-free Epstein-Barr virus DNA in plasma of patients with nasopharyngeal carcinoma. Cancer research. 1999;59(6):1188–91. Epub 1999/03/30. 10096545

[29] Pajek J, Kveder R, Gucek A, Skoberne A, Bren A, Bucar M, et al Cell-free DNA in the peritoneal effluent of peritoneal dialysis solutions. Therapeutic apheresis and dialysis: official peer-reviewed journal of the International Society for Apheresis, the Japanese Society for Apheresis, the Japanese Society for Dialysis Therapy. 2010;14(1):20–6. Epub 2010/05/05. 10.1111/j.1744-9987.2009.00717.x 20438516

[30] Che N, Yang X, Liu Z, Li K, Chen X. Rapid Detection of Cell-Free Mycobacterium tuberculosis DNA in Tuberculous Pleural Effusion. Journal of clinical microbiology. 2017;55(5):1526–32. Epub 2017/03/10. 10.1128/JCM.02473-16 28275073 PMC5405270

[31] Haldar S, Sankhyan N, Sharma N, Bansal A, Jain V, Gupta VK, et al Detection of Mycobacterium tuberculosis GlcB or HspX Antigens or devR DNA impacts the rapid diagnosis of tuberculous meningitis in children. PloS one. 2012;7(9):e44630 Epub 2012/09/18. 10.1371/journal.pone.0044630 22984534 PMC3440320

[32] Haldar S, Sharma N, Gupta VK, Tyagi JS. Efficient diagnosis of tuberculous meningitis by detection of Mycobacterium tuberculosis DNA in cerebrospinal fluid filtrates using PCR. Journal of medical microbiology. 2009;58(Pt 5):616–24. Epub 2009/04/17. 10.1099/jmm.0.006015-0 19369523

[33] Fernandez-Carballo BL, Broger T, Wyss R, Banaei N, Denkinger CM. Toward the Development of a Circulating Free DNA-Based In Vitro Diagnostic Test for Infectious Diseases: a Review of Evidence for Tuberculosis. Journal of clinical microbiology. 2019;57(4). Epub 2018/11/09. 10.1128/JCM.01234-18 PMC6440766 30404942

[34] Yang X, Che N, Duan H, Liu Z, Li K, Li H, et al Cell-free Mycobacterium tuberculosis DNA test in pleural effusion for tuberculous pleurisy: a diagnostic accuracy study. Clinical microbiology and infection: the official publication of the European Society of Clinical Microbiology and Infectious Diseases. 2020;26(8):1089 e1–e6. Epub 2019/12/06. 10.1016/j.cmi.2019.11.026 31805377

[35] Xuelian Li WD, Yuxuan Wang, Zichen Liu, Kun Li, Hongmei Chen, Rongmei Liu, et al Rapid Diagnosis of Tuberculosis Meningitis by Detecting Mycobacterium tuberculosis Cell-Free DNA in Cerebrospinal Fluid. Am J Clin Pathol. 2019;XX:1–5. 10.1093/ajcp/aqz135 31585003

[36] Shao L QC, Zheng L, Yang Y, Yang X, Liang Q, Zhang Y, et al Comparison of diagnostic accuracy of the GeneXpert Ultra, and cell-free nucleic acid assay for tuberculous meningitis: a multicenter prospective study. International Journal of Infectious Diseases. 2020; 10.1016/j.ijid.2020.06.076. 32599283

[37] Kumari P, Lavania S, Tyagi S, Dhiman A, Rath D, Anthwal D, et al A novel aptamer-based test for the rapid and accurate diagnosis of pleural tuberculosis. Analytical biochemistry. 2019;564–565:80–7. Epub 2018/10/24. 10.1016/j.ab.2018.10.019 30352198

[38] Van Hoving DJ, Griesel R, Meintjes G, Takwoingi Y, Maartens G, Ochodo EA. Abdominal ultrasound for diagnosing abdominal tuberculosis or disseminated tuberculosis with abdominal involvement in HIV-positive individuals. The Cochrane database of systematic reviews. 2019;9:CD012777 Epub 2019/10/01. 10.1002/14651858.CD012777.pub2 31565799 PMC6766789

[39] UNITAID. Tuberculosis Diagnostics Technology and Market Landscape, fourth ed. 2015.

[40] Rana S, Farooqui MR, Anees A, Ahmad Z, Jairajpuri ZS. The role of laboratory investigations in evaluating abdominal tuberculosis. Journal of family & community medicine. 2015;22(3):152–7. Epub 2015/09/24. 26392795 10.4103/2230-8229.163029 PMC4558736

[41] Horvath KD, Whelan RL. Intestinal tuberculosis: return of an old disease. The American journal of gastroenterology. 1998;93(5):692–6. Epub 1998/06/13. 10.1111/j.1572-0241.1998.207_a.x 9625110

[42] Bolognesi M, Bolognesi D. Complicated and delayed diagnosis of tuberculous peritonitis. The American journal of case reports. 2013;14:109–12. Epub 2013/07/05. 10.12659/AJCR.883886 23826447 PMC3700482

[43] Gyanchandani R, Kvam E, Heller R, Finehout E, Smith N, Kota K, et al Whole genome amplification of cell-free DNA enables detection of circulating tumor DNA mutations from fingerstick capillary blood. Scientific reports. 2018;8(1):17313 Epub 2018/11/25. 10.1038/s41598-018-35470-9 30470782 PMC6251935

[44] Augustus E, Van Casteren K, Sorber L, van Dam P, Roeyen G, Peeters M, et al The art of obtaining a high yield of cell-free DNA from urine. PloS one. 2020;15(4):e0231058 Epub 2020/04/07. 10.1371/journal.pone.0231058 32251424 PMC7135229

[45] Al Shohaib S. Tuberculous peritonitis in hemodialysis patients with chronic liver disease. Saudi journal of kidney diseases and transplantation: an official publication of the Saudi Center for Organ Transplantation, Saudi Arabia. 2000;11(4):577–82. Epub 2008/01/23. 18209348

[46] Chen HL, Wu MS, Chang WH, Shih SC, Chi H, Bair MJ. Abdominal tuberculosis in southeastern Taiwan: 20 years of experience. Journal of the Formosan Medical Association = Taiwan yi zhi. 2009;108(3):195–201. Epub 2009/03/19. 10.1016/S0929-6646(09)60052-8 19293034

[47] Elshimali YI, Khaddour H, Sarkissyan M, Wu Y, Vadgama JV. The clinical utilization of circulating cell free DNA (CCFDNA) in blood of cancer patients. International journal of molecular sciences. 2013;14(9):18925–58. Epub 2013/09/26. 10.3390/ijms140918925 24065096 PMC3794814

[48] Thierry AR, El Messaoudi S, Gahan PB, Anker P, Stroun M. Origins, structures, and functions of circulating DNA in oncology. Cancer metastasis reviews. 2016;35(3):347–76. Epub 2016/07/10. 10.1007/s10555-016-9629-x 27392603 PMC5035665

[49] Pisetsky DS, Fairhurst AM. The origin of extracellular DNA during the clearance of dead and dying cells. Autoimmunity. 2007;40(4):281–4. Epub 2007/05/23. 10.1080/08916930701358826 17516210

[50] Choi JJ RCI, Pisetsky DS. Release of DNA from dead and dying lymphocyte and monocyte cell lines in vitro. Scand J Immunol 2004;60:159 61. 10.1111/j.0300-9475.2004.01470.x 15238085

[51] Choi JJ RC III, Pisetsky DS. The role of macrophages in the in vitro generation of extracellular DNA from apoptotic and necrotic cells. Immunology. 2005:115–55. 10.1111/j.1365-2567.2005.02130.x PMC1782131 15819697

[52] Anatoli Kustanovich RS, Tamar Peretz, and Albert Grinshpun. Life and death of circulating cell-free DNA. Cancer Biology and Therapy. 2019;20(8):1057–67. 10.1080/15384047.2019.1598759 30990132 PMC6606043

[53] Leung F KV, Diamandis EP, et al Circulating tumor, 2016;62 DaacbfofCC, (8):1054–1060. Circulating tumor DNA as a cancer biomarker: fact or fiction? Clinical chemistry. 2016;62(8):1054–60. 10.1373/clinchem.2016.260331 27259816 PMC5326709

[54] Yu SCY LS, Jiang P, et al High-resolution profiling of fetal DNA clearance from maternal plasma by massively parallel sequencing. Clinical chemistry. 2013;59(8):1228–37. 10.1373/clinchem.2013.203679 23603797

[55] Lauber K BS, Waibel M, Wesselborg S. Lauber K1, Blumenthal SG, Waibel M, Wesselborg S. Mol Cell. 2004;14(3):277–87. 10.1016/s1097-2765(04)00237-0 15125832

[56] Fadok VA BD, Henson PM. Phagocyte receptors for apoptotic cells: recognition, uptake, and consequences. J Clin Invest. 2001;108(7):957–62. 10.1172/JCI14122 11581295 PMC200959

[57] Behar SM MC, Booty MG, Nishimura T, Zhao X, Gan HX, Divangahi M, et al Apoptosis is an innate defense function of macrophages against Mycobacterium tuberculosis. Mucosal immunology. 2011;4(3):279–87. 10.1038/mi.2011.3 21307848 PMC3155700

[58] Sharma SK, Kohli M, Chaubey J, Yadav RN, Sharma R, Singh BK, et al Evaluation of Xpert MTB/RIF assay performance in diagnosing extrapulmonary tuberculosis among adults in a tertiary care centre in India. The European respiratory journal. 2014;44(4):1090–3. Epub 2014/07/27. 10.1183/09031936.00059014 25063241

[59] Maynard-Smith L, Larke N, Peters JA, Lawn SD. Diagnostic accuracy of the Xpert MTB/RIF assay for extrapulmonary and pulmonary tuberculosis when testing non-respiratory samples: a systematic review. BMC infectious diseases. 2014;14:709 Epub 2015/01/21. 10.1186/s12879-014-0709-7 25599808 PMC4298952

