# Supplementary material for: Utility of circulating cell-free Mycobacterium tuberculosis DNA for the improved diagnosis of abdominal tuberculosis
Source: PLoS One. 2020 Aug 26;15(8):e0238119. doi: 10.1371/journal.pone.0238119 (PMC7449497; doi:10.1371/journal.pone.0238119)
Supplement: S1 Appendix — (PDF) [file pone.0238119.s001.pdf]

S1 APPENDIX
(CASE RECORD FORM)

Name : **Age :** **Sex : M / F**

CR No: **Date of admission :** **Date of discharge :**

Postal address: **Occupation :** **Phone :**

**History**	**1 if yes, 0 if No, Duration (days)**	**Examination**	**1 if yes, 0 if No**
Fever		Lympadenopathy	
Headache		Cold abscess	
Vomiting		Spine deformity	
Loss of appetite		Rash	
Constipation		Type of rash: (encircle)<br>Petechial /Pupuric /maculopapular /vescicular/other	
Diarrhoea			
Weight loss		Pallor/ Clubbing /I/Cy/JVP/PE	
Night Sweats		Any other finding on general physical examination:	
Abdominal pain			
Cough		Chest examination: RR/Trachea/Air entry/Adventitious sound	
Chest pain			
Seizure		CVS examination:<br>Heart sounds muffled : Yes/No	
Focal seizures			
HTN/CLD/CRF /CHF/RA/SLE		Worst GCS:	
DM		Decerebrate/decorticate posturing	
Hemoptysis		Focal deficits	
Altered sensorium		Extrapyramidal signs:<br>Type of deficit (encircle): Chorea, Dystonia, Tremor , Athetosis, Ballismus, Hemiparesis, Monoparesis, Aphasia, Blindness	
BCG Scar			
Contact with TB Patient			
Alcohol		Cranial Nerve involvement and details:	
Drug user history		Hepatomegaly	
Smoking		Splenomegaly	
Head injury/neurosurgery		Ascites	
Ear discharge		Any other significant finding	
Amenorrhoea			

Investigations:									
**Haemogram**	Hb		TLC		ESR		Platelet		
**DLC**									
**LFT**	D Bil		I Bil		AST		ALT		ALP
**KFT**	Urea		Creatinine		Uric Acid				
**Serum protein**	Total		Albumin		Globulin				
**Sputum AFB**									
**HIV/Cancer/Steroid therapy**									
**Mantoux**	**Pos.**				**Neg.**				

Fluid analysis (fill in relevant columns for each fluid type)	Pleural fluid	Ascitic fluid	CSF	Pericardial Fluid	Liver abscess fluid	Urine	Any Other fluid
Color							
Cytology :total cells/ml							
Cytology :differential							
Sugar							
Concomitant Blood Sugar (mg/dL)							
Protein (F+S)							
LDH (F+S)							
Albumin (F+S)							
SAAG ratio							
Light's criteria							
ADA							

Whether bacterial/viral/fungal isolation from other sites: Yes/ No   If yes, what site………………

**CT /MRI brain (tick √ if yes)**	Hydrocephalus	Basal exudates	TB Abscess	Ependymal/meningeal enhancement	Infarcts	Tuberculomas	Bacterial Abscess
**2D echo**	**Pre treatment**			**Post treatment**			
**CXR**	Effusion	Hazy lucencies	Miliary	Cavity	Consolidation	Collapse	Mediastinal LAP

Whether AFB found in other fluids: Yes/No

If yes, where: Sputum/Gastric aspirate/BAL/Pleural fluid/other……….

Whether FNAC/HPE of any tissue done: Y/ N

If yes, which tissue:…….

Finding: Inconclusive/ granulomas/ caseating granuloma/ other………………..

USG/CT abdomen if done: Ascites/LAP/ ileal thickening/ other findings………

Any other investigation done to support the diagnosis with findings:

Provisional Diagnosis:

Any other investigation:

Follow up:

ATT treatment: Yes/No, (If yes: then Duration ……....days); Weight Gain..................(yes / no)

Date of Start of ATT:………………   Date of Completion of ATT:……………

Any other test done to confirm follow up:

…………………………….
Signature of Senior Resident
Name:……………………..
